# Challenging Airway Management in Patients With Zenker’s Diverticulum

**DOI:** 10.7759/cureus.19578

**Published:** 2021-11-14

**Authors:** Christiano Dos Santos e Santos, Cristiane Araujo Tuma Santos, Lakshmi N Kurnutala

**Affiliations:** 1 Anesthesiology, University of Mississippi Medical Center, Jackson, USA; 2 Radiology, University of Mississippi Medical Center, Jackson, USA; 3 Anesthesiology and Perioperative Medicine, University of Mississippi Medical Center, Jackson, USA

**Keywords:** pulmonary aspiration, anesthesia, pharynx, zenker’s diverticulum, airway management

## Abstract

The acquired hypopharyngeal diverticulum (Zenker’s) is characterized by a posterior wall outpouching of the pharyngeal mucosa and submucosa through the vulnerable points of the pharyngoesophageal junction. We describe the case of a 67-year-old male who was recently diagnosed with Zenker's diverticulum and had complaints of dysphagia and halitosis. An endoscopic treatment (diverticulotomy) was performed without difficulties. The anesthetic management included rapid sequence induction, avoiding succinylcholine, and intraoperative infusion of dexmedetomidine. The neuromuscular blockade was reversed using sugammadex, decreasing the risk of failed extubation and possible airway re-intervention. The patient was discharged home the following day without complications.

## Introduction

The acquired hypopharyngeal diverticulum reported by Zenker and Von Ziemssen in 1874 is characterized by a posterior wall outpouching of the pharyngeal mucosa and submucosa through vulnerable points of the pharyngoesophageal junction [1,2]. Presumably, the upper esophageal sphincter's impaired function is correlated with Zenker's diverticulum pathophysiology [3]. The disease is regularly observed in elderly patients and frequently correlated with malnutrition [4]. The most frequent clinical manifestation of Zenker's diverticulum is dysphagia, while its dreadful consequence is pulmonary aspiration and subsequent pneumonitis [5]. 

Dynamic continuous fluoroscopy is usually the preferable investigation method to establish the diagnosis of Zenker's diverticulum [3]. The definitive treatment is cricopharyngeal myotomy that could be performed either via an open transcervical approach or endoscopy [6]. Currently, the most common procedure performed is the minimally invasive transoral flexible endoscopic stapling diverticulotomy [6].

The regurgitation to the glottis followed by bronchoaspiration remains one of the most concerning complications in the practice of anesthesiology. They include acute respiratory distress syndrome (ARDS), acute respiratory failure, and aspiration pneumonia [4]. Therefore, the American Society of Anesthesiologists provides recommendations for preoperative fasting and safe clinical anesthetic practice [7]. Nevertheless, patients with Zenker's diverticulum may present residual contents in the pharyngeal pouch for days (due to its putrified odor), implying a considerable risk of aspiration whose volume would be undeviatingly related to the size of the diverticulum. The traditional method of rapid sequence anesthetic induction with cricoid pressure is mainly practiced among anesthesiologists and emergency department physicians, thereby preventing pulmonary aspiration [8]. However, it could represent harmful consequences in this distinct sort of patient.

## Case presentation

**Figure 1:**
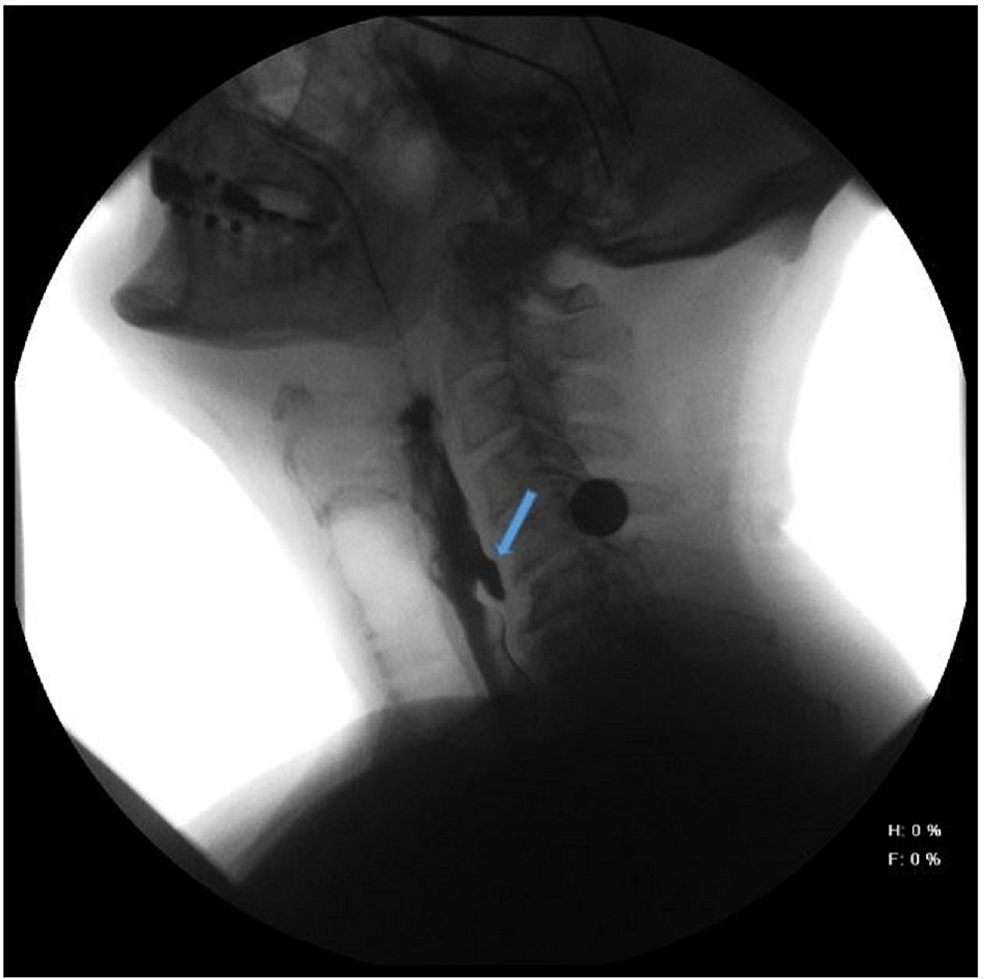
Video fluoroscopy showing hypopharyngeal triangular-shaped air pocket (blue arrow) adjacent to the esophagus with no visible fistula.

Our patient was a 67-year-old male with a past medical history of hypertension, atrial fibrillation, gout, obstructive sleep apnea, and recently diagnosed Zenker's diverticulum. He weighed 78 kg and was 182 cm in height (body mass index 23.5 kg/m2). All vital signs were in the normal range. His past surgical history included an uneventful bladder tumor removal four years ago, regular colonoscopy every five years since he turned fifty years old, and no previous anesthesia complications. He also denied using alcohol, tobacco, and recreational drugs. According to the patient, he went to see his primary care physician three weeks before this encounter, complaining of dysphagia and halitosis. The patient stated that dysphagia started approximately six weeks before his initial evaluation. He decided to see his primary care doctor because it was getting worse, and he assumed to have lost nearly five pounds during this period. Videofluoroscopy performed revealed the presence of a hypopharyngeal triangular-shaped air pocket adjacent to the esophagus with no visible fistula (Figure 1). He was currently taking allopurinol, lisinopril, omeprazole, atorvastatin, and metoprolol.

The patient and his wife were informed about the anesthetic plan and its risks in the preoperative ward. All questions and concerns were clarified. The consent was signed, and he was transported to the gastrointestinal suite/operating room. The patient's pre-induction vital signs were within normal limits. The American Society of Anesthesiologists monitors were installed, and a five-minute pre-oxygenation period was initiated. During this time, a loading dose of dexmedetomidine of 1 µg/kg was administered without any hemodynamic consequences, followed by the infusion of 0.6 µg/kg/hour during the entire procedure. After checking if suction was fully operating and available, the patient was placed in a semi-sitting (head 30 degrees elevated) position. A rapid sequence anesthetic induction was performed with lidocaine 1% (1 mg/kg), fentanyl (1 µg/kg), propofol (3 mg/kg), and rocuronium (1.2 mg/kg) without cricoid pressure. The endotracheal intubation (7.5 cuffed) was successfully performed via video-laryngoscope at the first attempt, without any sign of regurgitation or aspiration. Following the instrumentation of the airway, the patient's care was turned over to the gastroenterologist. No other pharmacologic treatments were necessary at that time, such as antibiotics or steroids. Anesthetic maintenance was achieved, continuing the infusion of dexmedetomidine and one minimal alveolar concentration (MAC) of sevoflurane. Before the diverticulotomy took place, a flexible esophagogastroduodenoscopy was conducted. During this phase, the content of the Zenker's diverticulum was aspirated and revealed a large amount of putrefied residual (110 mL). An unexceptional diverticulotomy was performed, and following the discontinuation of the infusion and volatile administration, the neuromuscular blocker agent was reversed via injection of sugammadex (2 mg/kg, based on the observation of the second twitch in response to a train of four stimulation).

The postoperative pain control was accomplished with hydromorphone 0.5 mg, and the patient was transferred from the post-anesthetic care unit to the surgical floor. A chest X-ray did not show any abnormality, and the patient was discharged home the subsequent day without complications.

## Discussion

The endotracheal intubation of an unconscious patient who could not protect their airway following anesthetic induction is the initial most vital goal in managing patients with a high risk of aspiration. Even though awake, airway instrumentation could be an alternative; this technique usually leads to the exacerbated irritation and stimulation of the pharynx that could culminate with a gag reflex, regurgitation, and consequent pneumonitis. 

Before anesthetic induction, an intentional self-induced regurgitation of residuals found in the diverticulum could be promoted [4]. The nasogastric tube placement or multiple intubation attempts are other unsafe choices considering the chance of diverticulum perforation and consequent mediastinitis [4]. Depending on the size of the diverticulum, its content can be significantly large, and the risk of pneumonitis increases exponentially.

The esophageal histological structure is constituted of smooth muscle in the distal portion, striated muscle in the proximal portion, and a mixture of both in the middle [9]. This peculiar muscular structure plays an essential role in the anesthetic plan. The fasciculation produced by succinylcholine could practically increase the intra-diverticular pressure, predisposing to regurgitation and aspiration. Therefore, we strongly suggest that succinylcholine would not be the best choice. Contrarily, the administration of rocuronium as a neuromuscular block agent for the rapid sequence would circumvent this possibility, being safer from our perspective. The use of dexmedetomidine is based on its pharmacological characteristic in reducing cough after general anesthesia with endotracheal intubation [10], which would predispose to surgical complications such as suture line rupture and consequent fistula formation. The safety of dexmedetomidine administration in patients with atrial fibrillation has been described in the literature [11].

The administration of sugammadex following local institution guidelines (2, 4, or 16 mg/kg, based on a train of four stimulation) [12] is crucial for preventing failed extubation and possible airway re-intervention. The re-intubation would contribute to an additional risk of perforation of the surgical site.

All patients should have a chest X-ray ordered in the postoperative period to rule out possible surgical complications such as pneumomediastinum or pneumothorax. A fistula to the mediastinum and consequent mediastinitis is a dreadful complication and needs to be addressed immediately due to its high mortality rate.

## Conclusions

Although Zenker's diverticulum is an uncommon disease (0.06%-4% found on endoscopic and radiologic studies), the anesthetic management of patients with this pathology is always challenging. Because of the risk of airway management difficulties, we strongly suggest that all anesthesiologists that work in gastrointestinal suites/thoracic surgery should be extra cautious when confronting patients with medium and large pouches. Additional help is encouraged and necessary for prompt intubation to decrease respiratory complications.
